# Anticoagulant for treatment and prophylaxis of venous thromboembolism patients with renal dysfunction: A systematic review and network meta-analysis

**DOI:** 10.3389/fmed.2022.979911

**Published:** 2022-09-26

**Authors:** Guohui Fan, Dingyi Wang, Meng Zhang, Xufei Luo, Zhenguo Zhai, Sinan Wu

**Affiliations:** ^1^Department of Clinical Research and Data Management, China-Japan Friendship Hospital, Beijing, China; ^2^National Center for Respiratory Disease, Beijing, China; ^3^National Clinical Research Center for Respiratory Disease, Beijing, China; ^4^Institute of Respiratory Medicine, Chinses Academy of Medical Sciences, Beijing, China; ^5^Department of Respiratory and Critical Care Medicine, Beijing Anzhen Hospital, Capital Medical University, Beijing, China; ^6^School of Public Health, Lanzhou University, Lanzhou, China; ^7^Department of Respiratory and Critical Care Medicine, China-Japan Friendship Hospital, Beijing, China

**Keywords:** anticoagulant agents, efficacy and safety, venous thromboembolism, renal insufficiency, network meta-analysis

## Abstract

**Objective:**

The aim of this study was to compare the efficacy and safety for particular regimen and dosage in venous thromboembolism (VTE) patients with renal insufficiency.

**Methods:**

English language searches of PubMed, Embase, and Web of Science (inception to May 2021). RCTs evaluating anticoagulants for VTE treatment at acute phase, extension phase, and VTE prophylaxis in patients with renal insufficiency and reporting efficacy (death, recurrence, or occurrence of VTE) and safety (bleeding) outcomes were selected. The methodological quality of each study included was assessed at the outcome level using the risk-of-bias assessment tool developed by the Cochrane Bias Methods Group.

**Results:**

Twenty-one trials that involved 76,574 participants and 8,972 (11.7%) patients with renal insufficiency were enrolled, including 10 trials on VTE treatment in acute phase (3–12 months), four trials on VTE treatment in extension phase (6–36 months), and seven trials for VTE prophylaxis. For acute VTE treatment, compared with dabigatran etexilate, apixaban (RR 5.90, 95%CI 1.00–34.60) and rivaroxaban (RR 6.18, 95%CI 1.17–32.75) were significantly associated with increased risk of death or recurrence. For extension treatment of VTE, aspirin had the highest probability of the most effective and safest treatment, followed by apixaban. For VTE prophylaxis, compared with enoxaparin, desirudin was associated with lower risk of VTE occurrence (RR 0.56, 95% CI 0.34–0.91), but had higher risk of bleeding than dabigatran etexilate.

**Conclusion:**

The network meta-analysis informs the optimal choice of anticoagulants and their particular dosage for treatment and prophylaxis of VTE patients comorbid renal insufficiency.

**Systematic review registration:**

www.crd.york.ac.uk/prospero/, identifier: CRD42021254086.

## Introduction

Chronic kidney disease (CKD)/renal insufficiency (RI) is associated with substantially increased risk for venous thromboembolism (VTE) (1, 2) and has been identified as an independent risk factor for short-term and long-term all-cause mortality and other adverse outcomes in VTE patients (3, 4). For example, compared with patients with normal renal function, patients with estimated glomerular filtration (eGFR) < 60 ml/min/1.73m^2^ had a 1.76-fold greater risk of short-term all-cause death, and patients with eGFR < 30 ml/min/1.73m^2^ had a 3.32-fold greater risk of short-term all-cause death and also higher rates of bleeding events (5).

Anticoagulation is the essential strategy for both VTE treatment and prophylaxis. Evidence-based guidelines recommended anticoagulation for the prevention of VTE in patients who have had major orthopedic or nonorthopedic surgery or hospitalized patients with acute illness (6), and for treatment of acute VTE (7). Unfractionated heparin (UFH), low molecular weight heparin (LMWH), and vitamin K antagonist (VKA) have been applied for decades. In recent years, direct oral anticoagulants (DOACs) addressed advantages over VKA and have been approved for both VTE treatment and prophylaxis worldwide.

Anticoagulation agents have proved their efficacy and safety by well-designed random controlled clinical trials (RCTs). Based on present evidence, dose adjustment recommendations for RI patients are for patients with creatinine clearance (CCr) ≤ 30 ml/min, LMWH is not recommended in European Society of Cardiology/European Respiratory Society (ESC/ERS) guideline, and anti-Xa levels need monitoring if LMWH was prescribed (7). Nevertheless, the usage and dose for DOACs for RI patients are incongruous in FDA recommendations and ESC/ERS guidelines (Supplementary Table 2). Since patients with severe RI were excluded in most clinical trials, drug/dose recommendation for those patients was insufficient. However, recent real-world studies revealed not only higher mortality rates, but also higher incidence of bleeding events in the treatment of VTE patients with RI (8–10), calling for an evaluation of the current administration of anticoagulation for those patients. Several meta-analyses have been conducted to compare different anticoagulation regimens among CKD/RI patients with both atrial fibrillation (AF) and VTE, but VTE patients with CKD/RI were seldom discussed. Particularly, recommended dosage of anticoagulants in treatment or prophylaxis of VTE was different from AF, especially in RI patients (Supplementary Table 2). Moreover, no meta-analysis emphasizing both categories and dosage of anticoagulants in VTE has been conducted. The aim of our systematic review and network meta-analysis was to evaluate the benefits and harms of different anticoagulants as well as their doses for treatment (both acute phase and extension use) and prophylaxis in VTE patients with RI.

## Methods

This systematic review and network meta-analysis were conducted according to the PRISMA Extension Statement for Network Meta-analysis (Preferred Reporting Items for Systematic reviews and Meta-Analysis) (11, 12). The study was registered with PROSPERO (CRD42021254086).

### Study eligibility and selection

We searched PubMed, Embase, and Web of Science from database inception up to 30 May 2021. We used keywords related to venous thromboembolism, kidney function, and anticoagulants in title and the full text of the articles. The full search strategies are provided in Supplementary Table 1. We hand searched reference lists from review articles and meta-analyses to identify any additional studies.

Two reviewers (W.D and F.G) independently performed the review, and disagreements were resolved in a panel discussion with an additional reviewer (W.S). The inclusion criteria for our study included (1) randomized controlled trials; (2) adult patients (≥18 years old) diagnosed with DVT and/or PE or required VTE prophylaxis; (3) treatment with intravenous or subcutaneous or oral anticoagulants (including DOACs, UFH, LMWH, VKA, and fondaparinux) compared with one another or placebo; (4) enrolled participants with determined RI; and (5) efficacy, bleeding outcomes, or both were reported. We excluded observational studies, crossover trials, patients with dialysis-dependent end-stage renal disease (ESRD), studies published in non-English language, and conference abstracts.

### Outcome measures

The primary efficacy outcomes were recurrent VTE or death associated with VTE for treatment trials and asymptomatic or symptomatic VTE or VTE-related death for prophylaxis trials. The safety outcomes were major bleeding and/or clinically relevant nonmajor bleeding according to the criteria in the International Society of Thrombosis and Haemostatsis (ISTH) (13).

### Data extraction and quality assessment

Data were extracted independently by two reviewers (W.D and F.G), and disagreements were resolved *via* consultation with another reviewer (W.S). A standardized form was used to extract the following data: study identifier, study design, location, length of follow-up, number of participants, age, sex, groups of renal function, intervention, and control details (drug name, dose, and timing); information relevant to the risk-of-bias assessment (including adherence to and withdrawal from randomized allocation); and definition of outcomes and number of events. The methodological quality of each included study was assessed at the outcome level independently by two reviewers (W.D and F.G) using the risk-of-bias assessment tool developed by the Cochrane Bias Methods Group (14) and checked by the third party (W.S). The risk-of-bias assessment tool includes seven aspects: random sequence generation (selection bias); allocation concealment (selection bias); blinding of participants and personnel (performance bias); blinding of outcome assessment (detection bias); incomplete outcome data (attrition bias); selective reporting (reporting bias); and other bias. Within each study, if one or more key domains were judged by reviewers as unknown risk or high risk, the study would be clarified as unknown risk or high risk. If the study was clarified as unknown risk and high risk at the same time, then high risk would be the final decision. As for the meta-analysis, whether studies at unknown or high risk of bias were sufficient to affect the interpretation of results depends on the proportion of these information.

### Data synthesis and analysis

As outcome data were acquired in different time points, we divided the studies into three categories (prophylaxis phase, acute phase, and extension phase). The data of each category were synthetized by standard meta-analysis and network meta-analyses within a frequentist environment, respectively, to simultaneously compare multiple regimens. Standard meta-analyses were used to analyze each pairwise direct comparison between interventions. Network meta-analysis combines evidence about treatments from direct head-to-head trials and indirectly from studies that used a common comparator for both treatments. The agreement between direct and indirect estimates in every closed loop of evidence using loop-specific and node-splitting approaches and global inconsistency test for the entire network using design-by-treatment interaction model was assessed. We assumed the same heterogeneity variance (τ^2^) for all comparisons in the network meta-analysis and compared its value with treatment-specific empirical distribution of variances to assess the magnitude of heterogeneity in the entire network. However, as the number of studies involved in each comparison was small, a fixed effect model was performed. Pooled relative risks (RRs) and 95% confidence (95% CIs) estimated for outcomes were binomial distribution using total number of patients randomized in each group as the denominator.

Network plots for comparisons were presented to visually describe the characteristics of involved studies. The nodes consisted in the network plots represent the treatments being compared, and edges represent the direct comparisons among different treatments. The sizes of nodes and the thicknesses of lines were weighted according to the sample size of specific comparison. Treatment strategies of DOACs, including doses and frequencies, for example, 2.5 mg twice daily or 5 mg once daily, were analyzed, respectively, and presented as independent dots in network plots. Studies without any events in all arms were excluded, and for those with at least one event in any arm, a constant of 0.5 was added to all cells in the table of that study. Cumulative rankograms for the network of adverse and safety outcomes were displayed to show the hierarchy for different interventions. The hierarchy was determined by the cumulative ranking probabilities estimated by network analysis for each treatment, which classified the competing treatments into first choice, second choice, …, etc.

Considering the competing treatments may be confusing, they were ranked according to their performance on one or more outcomes by clustered ranking plots. By using two-dimensional scaling approach, clustered ranking plots aimed to determine the optimal treatment for each efficacy/safety outcome group in each phase. Dendrograms of the hierarchical analysis were also displayed, which represent the dissimilarities between observations based on the cophenetic correlation coefficient and group the competing treatments into meaningful groups.

Publication bias was assessed using comparison-adjusted funnel plots for all active drugs against control. Statistical analyses were performed with Stata, version 16 (Stata, College Station, TX).

## Results

### Search results and characteristics of included studies

**Figure 1:**
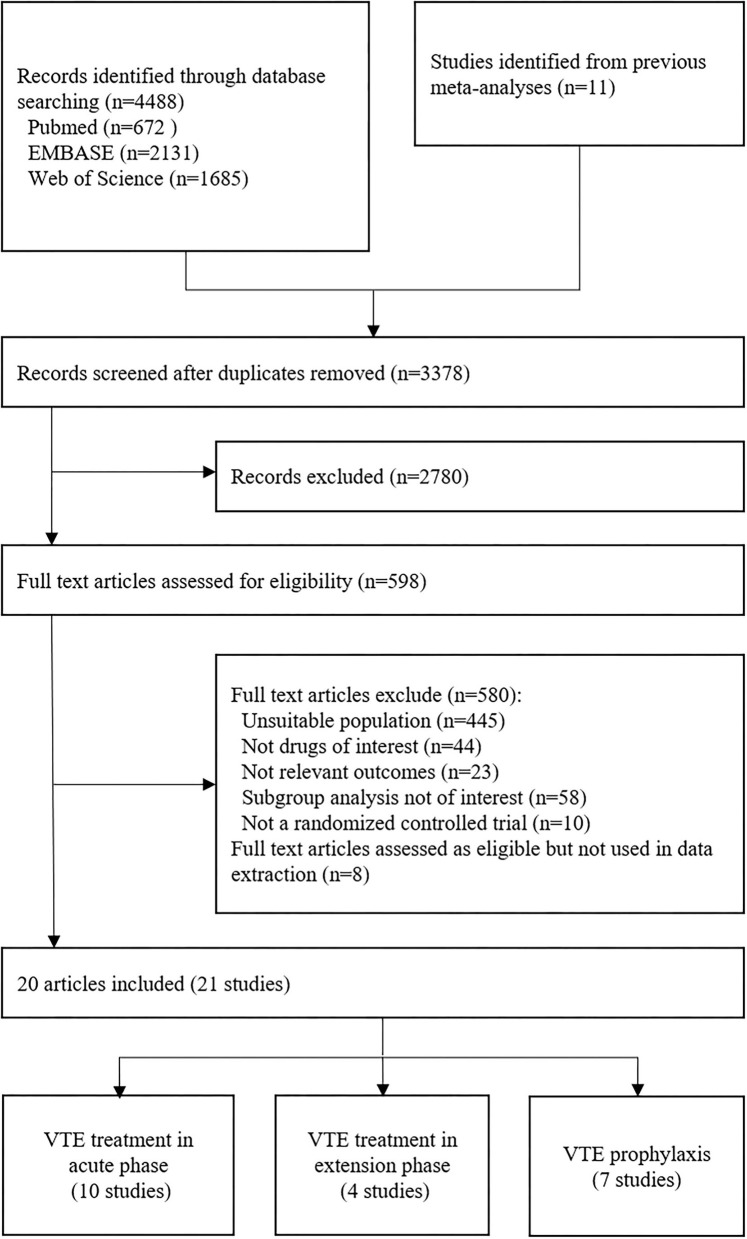
Flow diagram of the selection of involved studies.

Of the 21 trials we reviewed, 8,972 patients of total 76,574 participants had RI (see Tables 1, 2 to for a summary and details of trial characteristics). Ten trials, including 3,200 VTE patients with RI, were involved in acute phase (follow-up 3 to 12 months) (15–24). Four trials, involving 488 VTE patients with RI who completed 6 to 12 months anticoagulation therapy, were designed for comparison of extension anticoagulation therapies (follow-up 6 to 36 months) (22, 25–27). Seven trials, including 5,284 RI patients who were medically ill or underwent joint replacement surgeries or with cancer, were involved for VTE prophylaxis network analysis (follow-up 8 to 180 days) (28–34) (Figure 1).

**Table 1:** Summary of studies included in network meta-analysis.

**Study**	**Study population**	**Sample size**	**definition of RI**	**Renal-function based exclusion criteria**	**Number of patients with RI**	**intervention/ comparison**	**primary outcome (intervention/ comparison)**	**bleeding (intervention/ comparison)**
**VTE treatment in acute phase**								
RE-COVER-I and II (2017)	VTE	5,035	CCr < 50 ml/min	CCr < 30 ml/min	237	Dabigatran, 150 mg twice daily/VKA	0/5	21/29
CATCH (2018)	cancer associated VTE	864	eGFR < 60 ml/min/1.73m^2^	eGFR < 20 ml/min/1.73m^2^	131	Tinzaparin, 175I U/kg once daily/VKA	9/9	13/17
IRIS (2011)	>75 year old VTE	1,078	CCr < 60 ml/min	-	537	Tinzaparin, 175I U/kg once daily /UFH	8/4	13/17
Hokusai-VTE (2013)	VTE	8,240	CCr < 50 ml/min	CCr < 30 ml/min	541	Edoxaban, 30 mg once daily/VKA	8/15	32/32
Hokusai-VTE cancer (2018)	cancer associated VTE	1,046	CCr < 50 ml/min	CCr < 30 ml/min	72	Edoxaban, 30 mg once daily/Dalteparin, 200IU/kg once daily	2/1	4/1
CLOT (2016)	cancer associated VTE	676	CCr < 60 ml/min	-	162	Dalteparin, 193I U/kg once daily/VKA	2/15	15/21
AMPLIFY (2013)	VTE	5,365	CCr < 50 ml/min	CCr < 25 ml/min	539	Apixaban, 10 then 5 mg twice daily/enoxaparin, 1 mg/kg twice daily-VKA	7/7	5/9
AMPLIFY-cancer (2020)	cancer associated VTE	1,155	CCr < 80 ml/min	CCr < 25 ml/min	327	Apixaban, 10 then 5 mg twice daily /Dalteparin, 200I U/kg once daily	9/19	10/10
EINSTEIN-DVT (2010)	DVT	3,429	CCr < 50 ml/min	CCr < 30 ml/min	250	Rivaroxaban, 15 mg twice daily then 20 mg once daily/Enoxaparin-VKA	4/6	13/10
EINSTEIN-PE (2012)	PE	4,817	CCr < 50 ml/min	CCr < 30 ml/min	404	Rivaroxaban, 15 mg twice daily then 20 mg once /Enoxaparin-VKA	7/5	26/34
**VTE treatment in extension phase**								
EINSTEIN CHOICE (2017)	VTE treated 6 to 12 months	3,365	CCr < 50 ml/min	CCr < 30 ml/min	156	Rivaroxaban, 10 mg once daily/aspirin, 100 mg once daily	0/3/0	1/4/0
RE-MEDY and RE-SONATE (2013)	VTE treated in RECOVER I and II trials	4,199	CCr < 50 ml/min	-	108	Dabigatran, 150 mg twice daily/VKA	1/1/0/0	-
AMPLIFY-EXT (2013)	VTE treated 6 to 12 months	2,482	CCr < 50 ml/min	CCr < 25 ml/min	138	Apixaban, 5 mg or 2.5 mg twice daily/placebo	5/7	4/2/6
EINSTEIN extention (2010)	VTE treated 6 to 12 months	1,188	CCr < 50 ml/min	CCr < 30 ml/min	86	Rivaroxaban, 20 mg once daily/placebo	1/6	1/2
**VTE prophylaxis**								
MAGELLAN (2020)	≥40 years old, acute medical illness	7,998	CCr < 50 ml/min	CCr < 30 ml/min	1299	Rivaroxaban, 10 mg once daily/Enoxaparin, 40 mg once daily	9/15	36/17
MARINER (2018)	acute medical illness	11,962	CCr < 50 ml/min	CCr < 30 ml/min	2183	Rivaroxaban, 7.5mg once daily/placebo	18/18	20/10
Shorr (2012)	THR surgery	2,078	CCr < 60 ml/min	CCr < 30 ml/min	1006	Desirudin, 15 mg twice daily/Enoxaparin, 40 mg once daily	24/42	6/2
Dahl (2012)	joint replacement surgery	539	CCr < 50 ml/min	CCr < 30 ml/min	159	Dabigatran, 150 mg once daily/Enoxaparin, 40 mg once daily	3/8	0/6
ADVANCE-2 and 3 (2013)	THR surgery	6,788	CCr < 50 ml/min	CCr < 30 ml/min	318	Apixaban, 2.5mg twice daily/Enoxaparin, 40mg once daily	1/2	13/11
APEX (2016)	acute medical illness	3,429	CCr < 30 ml/min	CCr < 15 ml/min	256	Betrixaban, 80 mg-40 mg once daily/Enoxaparin, 20 mg once daily	12/10	3/1
CASSINI (2019)	ambulatory cancer patients with a higher risk of VTE	841	CCr < 50 ml/min	CCr < 30 ml/min	63	Rivaroxaban, 10 mg once daily/placebo	1/2	-

CCr, creatine clearance; PE, pulmonary embolism; RI, renal insufficiency; THR, total hip replacement; UFH, unfractionated heparin; VKA, vitamin K antagonist; VTE, venous thromboembolism.

**Table 2:** Direct comparisons and the rating the quality of evidence by the GRADE approach.

**Comparison**	**Treatment in acute phase**		**Treatment in extension phase**		**Prophylaxis for VTE**	
	**RR (95% CI)**	**GRADE**	**RR (95% CI)**	**GRADE**	**RR (95% CI)**	**GRADE**
tinzaparin, 1,75IU/kg, once VS. UFH 50IU/kg, twice	0.50 (0.07, 3.57)	High	No estimate	-	No estimate	-
tinzaparin, 1,75IU/kg, once VS. VKA	1.11 (0.19, 6.67)	High	No estimate	-	No estimate	-
dalteparin, 200IU/kg, once VS. VKA	6.25 (1.41, 25.00)	High	No estimate	-	No estimate	-
rivaroxaban VS. VKA	1.04 (0.26, 4.17)	High	No estimate	-	No estimate	-
rivaroxaban, 20 mg, once VS. aspirin, 100mg, once	No estimate	-	0.22 (0.01, 4.17)	High	No estimate	-
rivaroxaban, 10 mg, once VS. aspirin, 100mg, once	No estimate	-	0.18 (0.01, 3.38)	High	No estimate	-
rivaroxaban, 20 mg, once VS. placebo	No estimate	-	0.22 (0.03, 1.76)	High	No estimate	-
rivaroxaban, 10 mg, once VS. placebo	No estimate	-	No estimate	-	1.06 (0.57, 2.00)	Low
rivaroxaban, 10 mg, once VS. enoxaparin, 40 mg, once	No estimate	-	No estimate	-	1.61 (0.71, 3.70)	Low
apixaban, 10 mg, twice VS. VKA	1.08 (0.38,2.94)	Low	No estimate	-	No estimate	-
apixaban, 5 mg, twice VS. placebo	No estimate	-	0.69 (0.23, 2.00)	Low	No estimate	-
apixaban, 2.5 mg, twice VS. placebo	No estimate	-	0.15 (0.02, 1.17)	Low	No estimate	-
apixaban, 2.5 mg, twice VS. enoxaparin, 40 mg, once	No estimate	-	No estimate	-	1.92 (0.18, 20.00)	Low
apixaban, 10 mg, twice VS. dalteparin, 200IU/kg, once	2.38 (1.09, 5.26)	High	No estimate	-	No estimate	-
edoxaban, 30 mg, once VS. VKA	1.85 (0.23, 14.29)	Low	No estimate	-	No estimate	-
edoxaban, 30 mg, once VS. dalteparin, 200IU/kg, once	0.56 (0.03, 11.11)		No estimate	-	No estimate	-
dabigatran, 150 mg, twice VS. VKA	10.00 (0.38, 271)	Low	0.83 (0.05, 12.94)	Low	No estimate	-
dabigatran, 150 mg, twice VS. placebo	No estimate	-	0.73 (0.05, 11.24)	Low	No estimate	-
dabigatran 150 mg, once VS. enoxaparin, 40mg, once	No estimate	-	No estimate	-	2.08 (0.58, 7.69)	Low
betrixaban, 150 mg, once VS. enoxaparin, 40 mg, once	No estimate	-	No estimate	-	1.11 (0.50, 2.44)	Low
desirudin, 150mg, once VS. enoxaparin, 40 mg, once	No estimate	-	No estimate	-	1.79 (1.10, 2.94)	High

Direct comparisons were performed by standard meta-analyses.

CCr, creatine clearance; UFH, unfractionated heparin; VKA, vitamin K antagonist.

DOACs were compared with VKAs (six trials, 2,079 renal-insufficient patients), LMWH (six trials, 2,431 renal-insufficient patients), placebo (four trials, 2,470 renal-insufficient patients), and aspirin (one trial, 1,299 renal-insufficient patients), respectively. LMWH was compared with VKAs (two trials, 293 renal-insufficient patients), UFH (one trial, 537 renal-insufficient patients), and desirudin (one trial, 1,006 renal-insufficient patients), respectively. The funding source was reported in all trials, and all of them were sponsored by pharmaceutical companies (Table 1).

### Risks of bias and confidence rating

The risk-of-bias assessment for studies contributing to the network analyses of each outcome is presented in Table 2, Supplementary Figure 1. Most studies were assessed to be low risk for sequence generation and at low risk of bias for allocation concealment. Several studies were open-label and were assessed to be at high risk of bias for blinding of participants and investigators. Most studies were assessed to be at low risk of bias for blinding of outcome assessment and for incomplete outcome data.

### Effects of interventions

#### VTE treatment in acute phase

**Figure 2:**
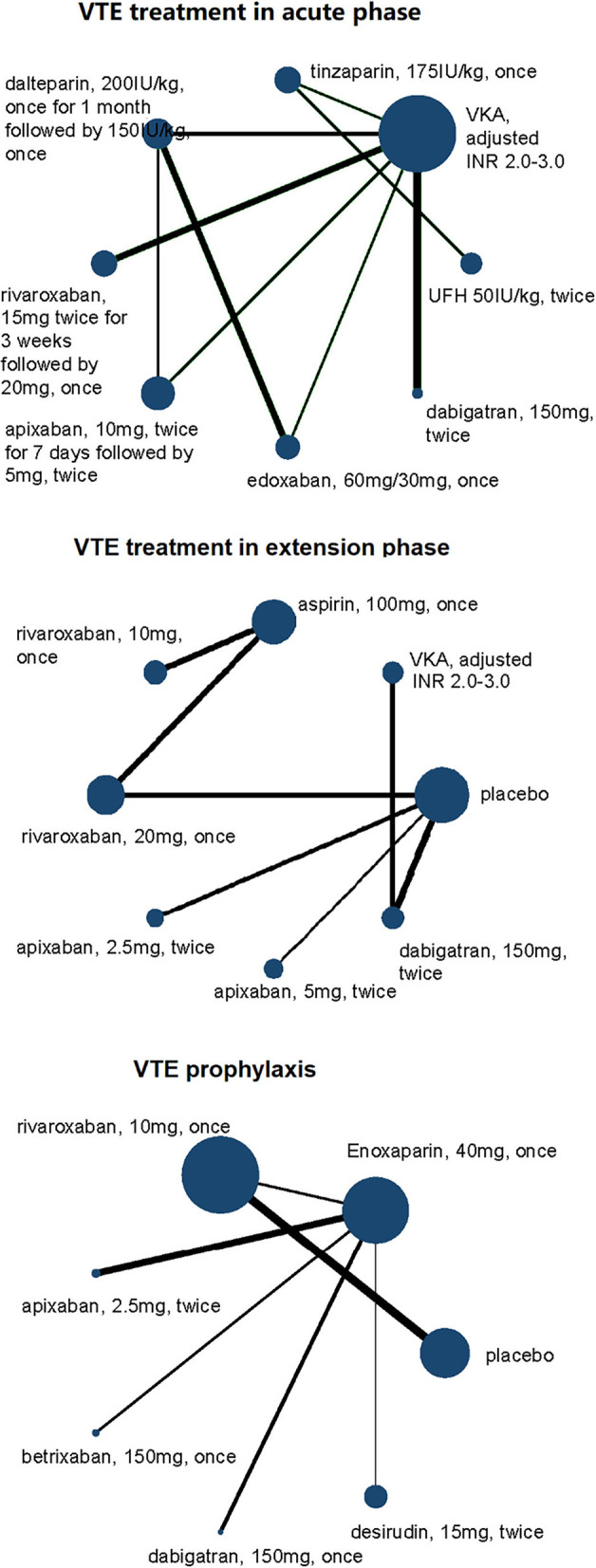
Network plots of VTE treatment network in acute phase, extension phase, and prophylaxis for efficacy outcomes of VTE patients with renal insufficiency. All drugs are present as daily dose and frequency. VTE, venous thrombus embolism; VKA, vitamin K antagonist.

**Figure 3:**
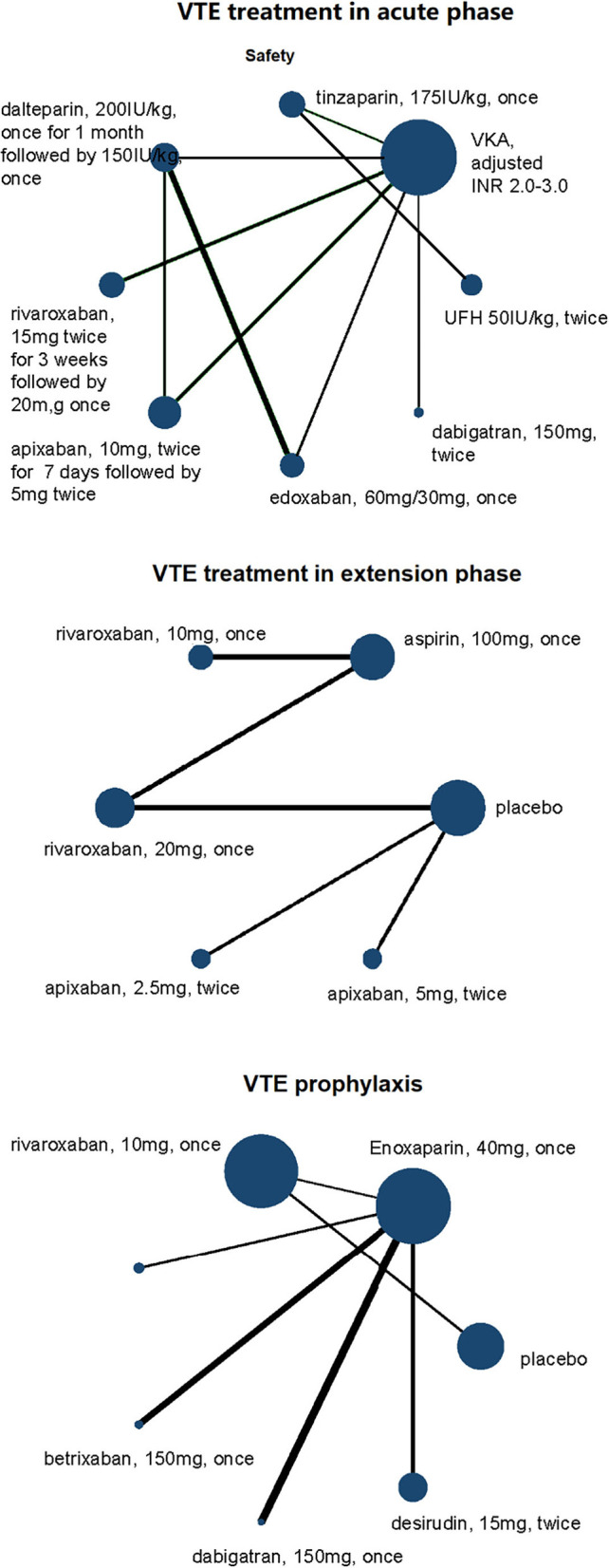
Network plots of VTE treatment network in acute phase, extension phase, and prophylaxis for safety outcomes of VTE patients with renal insufficiency. All drugs are present as daily dose and frequency. VTE, venous thrombus embolism; VKA, vitamin K antagonist.

Among the 10 studies focusing on acute phase, a total of 3,200 VTE patients with RI were involved. Seven of 10 studies took warfarin (adjusted INR 2.0–3.0) as controls, and other treatments and doses were UFH (50I U/kg, twice daily), tinzaparin (1,75I U/kg, once daily), dalteparin (2,00I U/kg, once daily), rivaroxaban (20 mg, once daily), apixaban (10 mg, twice daily), edoxaban (60 mg, once daily for normal renal function and 30 mg, once daily for RI patients), and dabigatran etexilate (150 mg, twice daily) (Figures 2, 3). Compared with dabigatran etexilate, apixaban (RR 5.90, 95% CI 1.00–34.60) and rivaroxaban (RR 6.18, 95% CI 1.17–32.75) were significantly associated with increased risk of death or recurrence in acute phase. Instead, compared with tinzaparin and warfarin, patients administered dalteparin may have lower risk (RR 0.18, 95% CI 0.03–0.95 for tinzaparin; RR 0.16, 95% CI 0.04–0.67 for warfarin) and apixaban may also reduce the risk than UFH (RR 0.07, 95% CI 0.01–0.49) (Table 3, Supplementary Figure 2).

**Table 3:** Network RR and 95%CI between different treatments in acute phase, extension phase, and prophylaxis for efficacy and safety outcomes of VTE patients with renal insufficiency.

**VTE treatment in acute phase**							
Dabigatran	0.96 (0.36, 2.55)	1.29 (0.42, 3.90)	0.87 (0.35, 2.17)	1.13 (0.42, 3.03)	1.13 (0.39, 3.27)	0.78 (0.39, 1.56)	1.13 (0.32, 3.96)
0.18 (0.01, 3.64)	Edoxaban	1.34 (0.44, 4.10)	0.91 (0.39, 2.11)	1.18 (0.43, 3.20)	1.18 (0.41, 3.38)	0.81 (0.41, 1.61)	1.17 (0.34, 4.10)
0.10 (0.00, 2.24)	0.58 (0.15, 2.19)	Apixaban	0.68 (0.23, 2.00)	0.88 (0.39, 1.97)	0.88 (0.27, 2.87)	0.61 (0.26, 1.44)	0.88 (0.23, 3.41)
0.10 (0.00, 2.01)	0.55 (0.17, 1.81)	0.95 (0.25, 3.58)	Rivaroxaban	1.30 (0.49, 3.40)	1.30 (0.48, 3.50)	0.89 (0.50, 1.60)	1.29 (0.39, 4.28)
0.62 (0.02, 15.54)	3.43 (0.64, 18.20)	5.90 (1.00, 34.60)	6.18 (1.17, 32.75)	Dalteparin	1.00 (0.35, 2.89)	0.69 (0.34, 1.38)	1.00 (0.28, 3.50)
0.11 (0.01, 2.21)	0.60 (0.18, 2.01)	1.04 (0.27, 3.96)	1.09 (0.33, 3.61)	0.18 (0.03, 0.95)	Tinzaparin	0.69 (0.31, 1.53)	1.00 (0.51, 1.95)
0.10 (0.01, 1.75)	0.54 (0.23, 1.26)	0.93 (0.34, 2.61)	0.98 (0.43, 2.26)	0.16 (0.04, 0.67)	0.90 (0.38, 2.12)	VKA	1.45 (0.51, 4.12)
0.52 (0.03, 9.37)	2.07 (0.35, 12.40)	0.07 (0.01, 0.49)	2.17 (0.40, 11.75)	0.35 (0.04, 2.75)	1.99 (0.61, 6.54)	2.22 (0.51, 9.60)	UFH
**VTE treatment in extension phase**							
Dabigatran	NA	NA	NA	NA	NA	NA	NA
7.30 (0.23, 230.33)	Apixaban, 5mg	0.60 (0.06, 5.72)	2.89 (0.16, 51.59)	8.13 (0.08, 825.86)	1.13 (0.03, 41.26)	NA	1.92 (0.37, 9.97)
33.48 (0.70, 1604.74)	4.58 (0.45, 46.54)	Apixaban, 2.5mg	4.85 (0.29, 81.54)	13.62 (0.14, 1334.22)	1.89 (0.05, 65.97)	NA	3.21 (0.68, 15.05)
22.65 (0.47, 1097.12)	3.10 (0.30, 32.04)	0.68 (0.04, 12.53)	Rivaroxaban, 20mg	2.81 (0.08, 104.15)	0.39 (0.05, 3.37)	NA	0.66 (0.06, 7.03)
28.05 (0.10, 8267.39)	3.84 (0.03, 451.71)	0.84 (0.01, 134.56)	1.24 (0.02, 79.06)	Rivaroxaban, 10mg	0.14 (0.01, 2.52)	NA	0.24 (0.00, 17.65)
5.01 (0.04, 650.58)	0.69 (0.02, 29.23)	0.15 (0.00, 9.41)	0.22 (0.01, 4.17)	0.18 (0.01, 3.38)	Aspirin	NA	1.70 (0.07, 41.54)
0.76 (0.05, 11.87)	0.10 (0.00, 8.58)	0.02 (0.00, 2.62)	0.03 (0.00, 3.90)	0.03 (0.00, 15.02)	0.15 (0.00, 40.66)	VKA	NA
5.00 (0.19, 132.83)	0.68 (0.23, 2.00)	0.15 (0.02, 1.16)	0.22 (0.03, 1.76)	0.18 (0.00, 18.55)	1.00 (0.03, 36.38)	6.56 (0.09, 471.92)	Placebo
**VTE prophylaxis**							
Desirudin	28.93 (1.09, 768.32)	1.15 (0.07, 18.22)	2.41 (0.41, 14.26)	1.34 (0.25, 7.32)	2.96 (0.60, 14.61)	2.69 (0.42, 17.17)	
1.17 (0.30, 4.66)	Dabigatran	0.04 (0.00, 1.52)	0.08 (0.00, 1.62)	0.05 (0.00, 0.86)	0.10 (0.01, 1.79)	0.09 (0.00, 1.90)	
0.62 (0.24, 1.58)	0.53 (0.12, 2.41)	Betrixaban	2.09 (0.19, 22.71)	1.17 (0.11, 11.91)	2.57 (0.27, 24.44)	2.33 (0.20, 26.82)	
1.08 (0.09, 12.37)	0.92 (0.06, 13.88)	1.74 (0.14, 21.67)	Apixaban	0.56 (0.21, 1.46)	1.23 (0.56, 2.68)	1.11 (0.33, 3.79)	
0.90 (0.35, 2.33)	0.76 (0.17, 3.52)	1.45 (0.46, 4.56)	0.83 (0.07, 10.42)	Rivaroxaban	2.20 (1.25, 3.88)	2.00 (0.94, 4.25)	
0.56 (0.34, 0.91)	0.48 (0.13, 1.73)	0.90 (0.41, 2.02)	0.52 (0.05, 5.67)	0.62 (0.27, 1.41)	Enoxaparin	0.91 (0.35, 2.33)	
0.85 (0.27, 2.64)	0.72 (0.14, 3.75)	1.36 (0.37, 5.03)	0.78 (0.06, 10.58)	0.94 (0.50, 1.76)	1.51 (0.54, 4.23)	Placebo	

White background (bottom-left): results of efficacy outcome; gray background (top-right): results of safety outcome.

VTE, venous thrombus embolism; RR, risk ratio; 95%CI, 95% confidence interval; VKA, vitamin K antagonist. The pink color refers to the anticoagulants administered in the trial.

**Figure 4:**
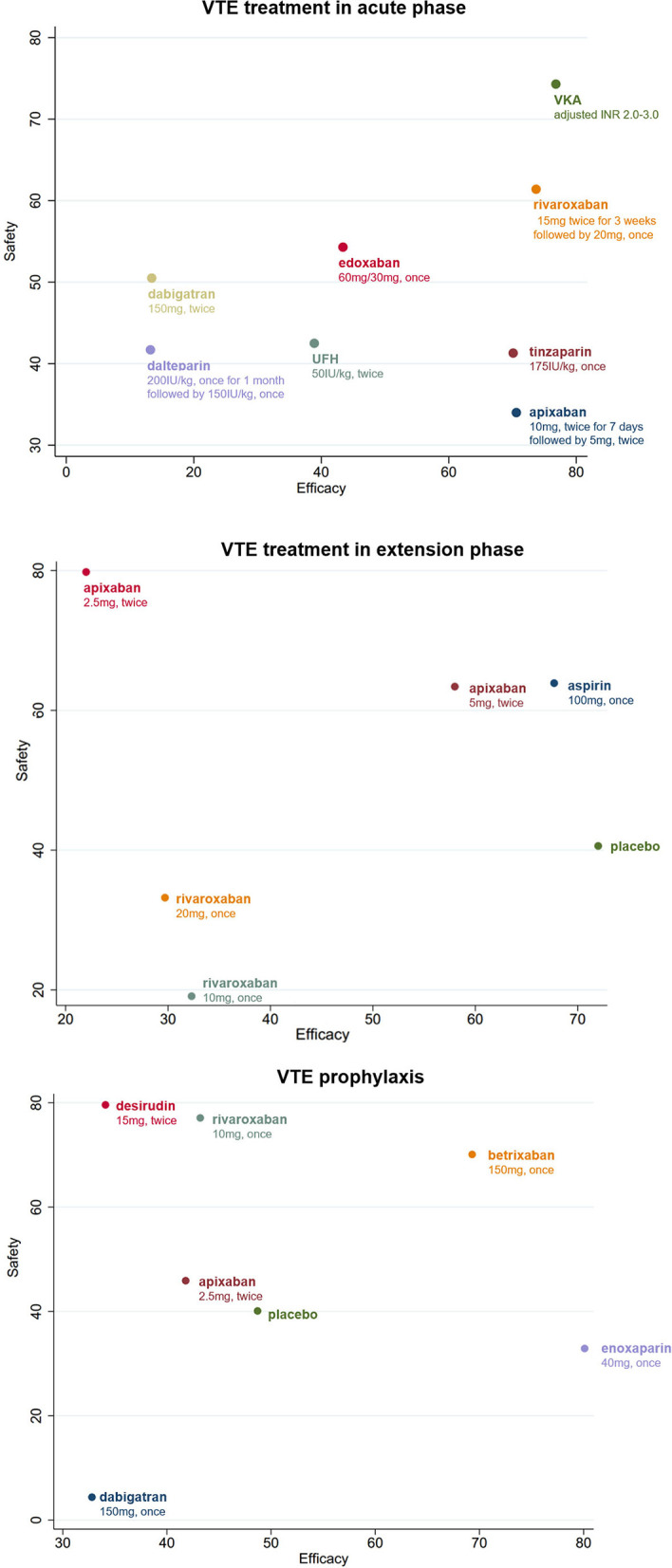
Clustered ranking plot of the different treatments based on cluster analysis for efficacy and safety for VTE patients with renal insufficiency. All drugs are present as daily dose and frequency. VTE, venous thrombus embolism; VKA, vitamin K antagonist.

Clustered ranking plots indicated that warfarin had the highest probability of the most effective and safest treatment than the other treatments in acute phase, and the secondary treatment was rivaroxaban (Figure 4, Supplementary Figures 3, 4).

#### VTE treatment in extension phase

A total of eight different treatments from four studies with 488 VTE patients with RI involved in the extension phase network analysis and the direct comparisons of efficacy and safety are shown in Figures 2, 3. However, no evidence in direct comparison or network comparison indicated that any treatment would reduce or increase VTE risk in this phase (Tables 2, 3, Supplementary Figure 2). According to the clustered ranking plots, aspirin had the highest probability of the most effective and safest treatment than the other treatments in extension phase, followed by apixaban (Figure 4, Supplementary Figures 3, 4).

#### VTE prophylaxis

The network analysis of VTE prophylaxis phase contains seven different treatments from seven studies involving 5,284 VTE patients with RI. The direct comparisons of efficacy and safety are shown in Figures 2, 3. Compared with enoxaparin, desirudin was associated with lower risk of VTE occurrence (RR 0.56, 95% CI 0.34–0.91); however, it significantly increased the risk of bleeding compared with dabigatran etexilate (RR 28.93, 95% CI 1.09–768.32). The risk of bleeding in VTE prophylaxis was lower for patients administered with dabigatran etexilate than those with rivaroxaban (RR 0.05, 95% CI 0.00–0.86) and was higher for those with rivaroxaban than with enoxaparin (RR 2.20, 95% CI 1.25–3.88), although the efficacy was comparable among these treatments (Tables 2, 3, Supplementary Figure 2). The clustered ranking plots show that betrixaban may have the highest probability of the most effective and safest treatment than the other treatments in prophylaxis phase (Figure 4, Supplementary Figures 3, 4).

Asymmetries of the funnel plots of network analysis for VTE patients with RI were acceptable and suggested few presence of small-study effects (Supplementary Figure 5).

Considering their treatment which may differ from VTE patients with renal insufficiency, those without RI were also involved in another independent network analysis. For VTE patients without RI, seven, eight, and five treatments were found in the network analyses of acute phase, extension phase, and prophylaxis phase, respectively (Supplementary Figure 6). The treatment with highest probability of the most effective and safest treatment than the other treatments in acute phase was warfarin (adjusted INR 2.0–3.0), in extension phase was rivaroxaban (20 mg, once daily), and in prophylaxis phase was enoxaparin (40 mg, once daily). More details and results are presented in Supplementary Figures 7–11.

## Discussion

Our analysis indicates that for patients with RI, currently available anticoagulant with the highest probability of the most effective and safest treatment was VKAs in VTE treatment during acute phase, aspirin in VTE extension treatment, followed by apixaban, and betrixaban in VTE prophylaxis, followed by enoxaparin. To the best of our knowledge, this is the first and most comprehensive network meta-analysis focusing on the efficacy and safety of anticoagulation regimens and their particular dosage on VTE patients comorbid RI.

There is no current guideline or recommendation that indicates whether one anticoagulant is preferred for VTE patients with RI over another. Previous meta-analysis on applications of anticoagulants among CKD patients: Harel et al. compared DOACS and VKA in 4 RCTs and found that neither efficacy nor safety outcomes among VTE patients with CKD were different between the two specimens of anticoagulants (35). Ha et al. evaluated the benefits and harms of VKAs and DOACs in patients with CKD stages 3–5, including those with dialysis-dependent ESRD reported no significant difference in either efficacy or safety outcomes, between DOACs and LMWH, DOACs and VKAs, and VKAs and LMWH (35, 36). They also showed that in patients with AF and early-stage CKD, DOACs were superior to VKAs, with significant reduction of systemic embolism and bleeding events. However, for VTE patients with CKD, the advantages of DOACs compared with VKAs were uncertain and controversial (37). In our network meta-analysis, we sorted anticoagulant regimens as particular species and dosage, other than by pharmacological mechanisms (DOACs and LMWH), for the purpose of an identification of more exact recommendation of anticoagulation for either VTE treatment or prophylaxis among patients with RI. In real-world settings, patients with RI (especially severe RI) or CKD, the complexity of comorbidity and co-medication increases the difficulty of adjustment and monitoring the dose of VKA, which might be a reason of the result from some observational studies that a higher risk of bleeding occurred among CKD patients administered with VKA (38).

CKD was observed 10.7% in the Chinese population and has been increasing along with the high prevalence of diabetes and hypertension (39, 40). Folsom et, al revealed a 1.6- to 1.7-fold risk for CKD stage 3–4 patients to develop VTE (41), and the prevalence of RI among PE patients was around 27% to 49% (42) 43, 44. Recent evidence indicates higher rates of bleeding events among those patients (10, 42). Lim et, al revealed a significantly higher risk of major bleeding in patients with a CCr ≤ 30 ml/min than those with a CCr >30 ml/min in a meta-analysis. The risk of major bleeding was increased when a standard therapeutic dose of enoxaparin was used, but not when an empirically adjusted dose of enoxaparin was used [45]. Researchers recently rose the point of the influence of anticoagulation therapy to bleeding events in patients with CKD or RI [9, 46]. Pharmacokinetic studies also suggested an association between creatinine clearance and levels of antifactor Xa heparin, and guidelines recommended the Xa level should be monitored during LMWH application in RI patients, which needed wide application in clinical practice [47].

In our study, we also conducted network analysis among patients with normal renal function. The results of preference were different from RI patients, either in acute, extension phase, or VTE prophylaxis. These results emphasized the importance of different considerations in choosing appropriate anticoagulation regimens according to the renal function of VTE patients. During the analysis, we recognized that the number of RI patients was far less than those without RI, and those with severe RI (e.g., CCr or eGFR < 15 or 30 ml/min) were excluded from the design of the RCTs. Thus, recommendations of certain dosage for RI patients would be weak, and even absent for patients with severe RI. The small sample size of patients was one of the reasons of potential heterogeneity of the results. Two highly anticipated ongoing studies were focused on patients with AF and ESRD, RENAL-AF (RENal Hemodialysis Patients ALlocated Apixaban Versus Warfarin in Atrial Fibrillation) (Clinical Trials.gov: NCT02942407), and AXADIA (ClinicalTrials.gov: NCT02933697), but for VTE patients with CKD/RI, there still requires studies providing stronger evidence for certain recommendation of anticoagulation therapy.

The study has several limitations. First, VTE patients with RI in each study were much less in number than those without RI and were different in comorbidities, like cancer, and post-hip or knee replacement, which inevitably became the possible source of heterogeneity. The variability in definition of RI among involved studies, which varies from CCr < 50 ml/min to CCr < 80 ml/min, also impacted the potential variability in source populations. Both sample size and various definitions of RI may lead to the inaccurate estimation of pooled RRs, which were noted by wide confidence intervals and extreme effect estimates. Second, the network may be incomplete in some network analyses for the competing treatments which was classified by their regimens and dosages. For example, there was only one study focused on aspirin and UFH. Therefore, the global inconsistency test for those entire networks would be inaccurate, and only consistency models were performable. Third, the heterogeneity between the studies might affect the results of our study. For example, some studies that focused on cancer patients would have higher rates of all-cause mortality than other studies. Similarly, for the prophylaxis purpose, different patients were under different risks of VTE (e.g., medically ill and surgery), thus leading to difference of occurrence. Analysis for specific group of patients is required after more studies published in future. Fourth, even though the funnel plot was symmetrical, there were some studies focused on anticoagulation treatment and VTE patients of various renal functions were excluded due to unavailable data in patients with RI. These articles may contribute some publication bias to our study. Fifth, the grades of ROBs differ among different included trials, because in some studies, the anticoagulants were administered by i.v. or oral delivery. Therefore, it would be impossible to blind. However, our study highlights the absence of evident in patients with RI, especially for patients who require treatment or prevention of VTE. The potential benefit of anticoagulation needs to be weighed against the risk for bleeding in this population.

## Conclusion

The network meta-analysis informs the optimal choice of anticoagulants and their particular dosage for treatment and prophylaxis of VTE patients comorbid RI. Studies designed for patients with RI are required for more sufficient evidence for establishing benefits and harms for anticoagulants.

## Data availability statement

The original contributions presented in the study are included in the article/Supplementary material, further inquiries can be directed to the corresponding author.

## Author contributions

GF, DW, and SW designed the study and drafted the manuscript. GF and DW searched the database and screened the involved studies. GF, DW, and XL analyzed the data. GF, DW, MZ, XL, ZZ, and SW revised the manuscript and approved it for submission. All authors contributed to the article and approved the submitted version.

## Funding

This study was funded by the National High Level Hospital Clinical Research Funding (2022-NHLHCRFLX-01-01-01), Fundamental Research Funds for the Central Universities (3332021086), and China-Japan Friendship Hospital Grant (2019-2-QN-78).

## Conflict of interest

The authors declare that the research was conducted in the absence of any commercial or financial relationships that could be construed as a potential conflict of interest.

## Publisher's note

All claims expressed in this article are solely those of the authors and do not necessarily represent those of their affiliated organizations, or those of the publisher, the editors and the reviewers. Any product that may be evaluated in this article, or claim that may be made by its manufacturer, is not guaranteed or endorsed by the publisher.
