# Supplementary material for: Anticoagulant for treatment and prophylaxis of venous thromboembolism patients with renal dysfunction: A systematic review and network meta-analysis
Source: Front Med (Lausanne). 2022 Sep 26;9:979911. doi: 10.3389/fmed.2022.979911 (PMC9548609; doi:10.3389/fmed.2022.979911)
Supplement: Supplementary file 1 [file Data_Sheet_1.docx]

Supplementary Table 1 Full search strategies for published studies

| Pubmed |
| --- |
| (anticoagulant*[Title/Abstract] OR anticoagulation*[Title/Abstract] OR LMWH[Title/Abstract] OR VKA[Title/Abstract] OR NOAC*[Title/Abstract] OR DOAC*[Title/Abstract] OR UFH[Title/Abstract] OR low molecular weight heparin[Title/Abstract] OR unfractionated heparin[Title/Abstract] OR warfarin[Title/Abstract] OR direct oral anticoagulant*[Title/Abstract] OR novel[Title/Abstract] OR anticoagulant*[Title/Abstract] OR dabigatran[Title/Abstract] OR rivaroxaban[Title/Abstract] OR apixaban[Title/Abstract] OR edoxaban[Title/Abstract] OR enoxaparin[Title/Abstract] OR dalteparin[Title/Abstract] OR tinzaparin[Title/Abstract] OR fondaparinux[Title/Abstract] OR argatroban[Title/Abstract])  AND  (venous thromboembo*[Title/Abstract] OR pulmonary embo*[Title/Abstract] OR pulmonary thromboembo*[Title/Abstract] OR VTE[Title/Abstract] OR PE[Title/Abstract] OR PTE[Title/Abstract])  AND  (chronic kidney disease[Title/Abstract] OR renal failure[Title/Abstract] OR renal impairment[Title/Abstract] OR end stage renal disease[Title/Abstract] OR end-stage renal disease[Title/Abstract] OR dialysis[Title/Abstract] OR kidney failure[Title/Abstract] OR kidney insufficiency[Title/Abstract] OR kidney dysfunction[Title/Abstract] OR renal insufficiency[Title/Abstract] OR renal dysfunction[Title/Abstract] OR CKD[Title/Abstract] OR ESRD[Title/Abstract] OR ESKD[Title/Abstract]) |
| EMBASE |
| ((anticoagulant*):ab,ti OR (anticoagulation*):ab,ti OR (LMWH):ab,ti OR (VKA):ab,ti OR (NOAC*):ab,ti OR (DOAC*):ab,ti OR (UFH):ab,ti OR (low molecular weight heparin):ab,ti OR (unfractionated heparin):ab,ti OR (warfarin):ab,ti OR (direct oral anticoagulant*):ab,ti OR (novel):ab,ti OR (anticoagulant*):ab,ti OR (dabigatran):ab,ti OR (rivaroxaban):ab,ti OR (apixaban):ab,ti OR (edoxaban):ab,ti OR (enoxaparin):ab,ti OR (dalteparin):ab,ti OR (tinzaparin):ab,ti OR (fondaparinux):ab,ti OR (argatroban):ab,ti )  AND  ((venous thromboembo*):ab,ti OR (pulmonary embo*):ab,ti OR (pulmonary thromboembo*):ab,ti OR (VTE):ab,ti OR (PE):ab,ti OR (PTE):ab,ti )  AND  ((chronic kidney disease):ab,ti OR (renal failure):ab,ti OR (renal impairment):ab,ti OR (end stage renal disease):ab,ti OR (end-stage renal disease):ab,ti OR (dialysis):ab,ti OR (kidney failure):ab,ti OR (kidney insufficiency):ab,ti OR (kidney dysfunction):ab,ti OR (renal insufficiency):ab,ti OR (renal dysfunction):ab,ti OR (CKD):ab,ti OR (ESRD):ab,ti OR (ESKD):ab,ti ) |
| WebofScience |
| TS=(anticoagulant* OR anticoagulation* OR LMWH OR VKA OR NOAC* OR DOAC* OR UFH OR low molecular weight heparin OR unfractionated heparin OR warfarin OR direct oral anticoagulant* OR novel OR anticoagulant* OR dabigatran OR rivaroxaban OR apixaban OR edoxaban OR enoxaparin OR dalteparin OR tinzaparin OR fondaparinux OR argatroban)  AND  TS= (venous thromboembo* OR pulmonary embo* OR pulmonary thromboembo* OR VTE OR PE OR PTE)  AND  TS= (chronic kidney disease OR renal failure OR renal impairment OR end stage renal disease OR end-stage renal disease OR dialysis OR kidney failure OR kidney insufficiency OR kidney dysfunction OR renal insufficiency OR renal dysfunction OR CKD OR ESRD OR ESKD) |

**Supplementary Table 2. Summary of current recommendations for VTE patients with RI from guidelines or FDA recommendations**

| **Drug Name** | **Indication** | **Renal function group** | **Recommend dose for renal impairment** | **Reference** | **Published date** |  |
| --- | --- | --- | --- | --- | --- | --- |
| Betrixazban | prophylaxis of VTE | CCr≥15 to <30ml/min | 80mg once daily followed by 40mg once daily for 35 to 42 days | FDA orange book | 06/2017 |  |
|  |  |  |  |  |  |  |
| Rivaroxaban | Treatment of DVT and/or PE | CCr<15ml/min | Avoid use | FDA orange book | 01/2020 |  |
|  |  |  |  |  |  |  |
|  | Reduction in the risk of recurrence of DVT and/or PE in patients at continued risk for DVT and/or PE | CCr<15ml/min | Avoid use |  |  |  |
|  |  |  |  |  |  |  |
| Delteparin Sodium | extended treatment of acute symtomatic VTE in adult patients with cancer | CCr<30ml/min | Monitor anti-Xa levels (target anti-Xa range 0.5-1.5 IU/ml) | FDA orange book | 12/2020 |  |
| Enoxaparin Sodium | prophylaxis of VTE | CCr<30ml/min | 30mg once daily | FDA orange book | 12/2018 |  |
|  | Treatment of DVT and/or PE | CCr<30ml/min | 1mg/kg once daily |  |  |  |
| Warfarin Sodium | Prophylaxis and treatment of venous thrombosis and its extension,pulmonary embolism | renal impairment | no dosage adjustment but need to monitor INR more frequently | FDA orange book | 05/2017 |  |
| Apixaban | Prophylaxis of Deep Vein Thrombosis Following Hip or Knee Replacement Surgery, and treatment of DVT and PE and Reduction in the Risk of Recurrence of DVT and PE | renal impairment, including those with ESRD on dialysis | no dose adjustment | FDA orange book | 04/2021 |  |
|  | Treatment for acute PE | CCr<15ml/min | not recommended | 2019 ESC/ERS guideline for acute PE | 2019 |  |
| Dabigatran Etexilate | treatment and reduction in the risk of recurrence of DVT and PE | CCr<50ml/min | aviod use | FDA orange book | 07/2020 |  |
|  | Prophylaxis of DVT and PE following hip replacement surgery | CCr<50ml/min | aviod use |  |  |  |
|  | Treatment for acute PE | Concomitant treatment with P-gp inhibiors in patients with CCr<50ml/min | not recommended | 2019 ESC/ERS guideline for acute PE | 2019 |  |
|  |  | CCr <30ml/min | not recommended |  |  |  |
| Edoxaban | treatment of DVT and PE following 5 to 10 days of initial therapy with a parenteral anticoagulant | CCr 15-50 ml/min | 30mg once daily | FDA orange book | 09/2017 |  |
|  |  | CCr<15ml/min | no recommendation |  |  |  |
|  | Treatment for acute PE | CCr<15ml/min | not recommended | 2019 ESC/ERS guideline for acute PE | 2019 |  |

Abbreviations: CCr, creatine clearance; DVT, deep vein thrombosis; ERS, European respiratory society; ESC, European society of cardiology; ESRD, end-stage renal disease; FDA, food and drug administration; PE, pulmonary embolism; RI, renal insufficiency; UFH, unfractionated heparin; VKA, vitamin K antagonist; VTE, venous thromboembolism.

**Supplementary Figure 1. Classification of risk of bias for each study domain among VTE patients with based on Cochrane tool.**

Abbreviations:
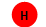
, high risk;
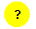
, unclear risk;
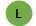
, low risk.


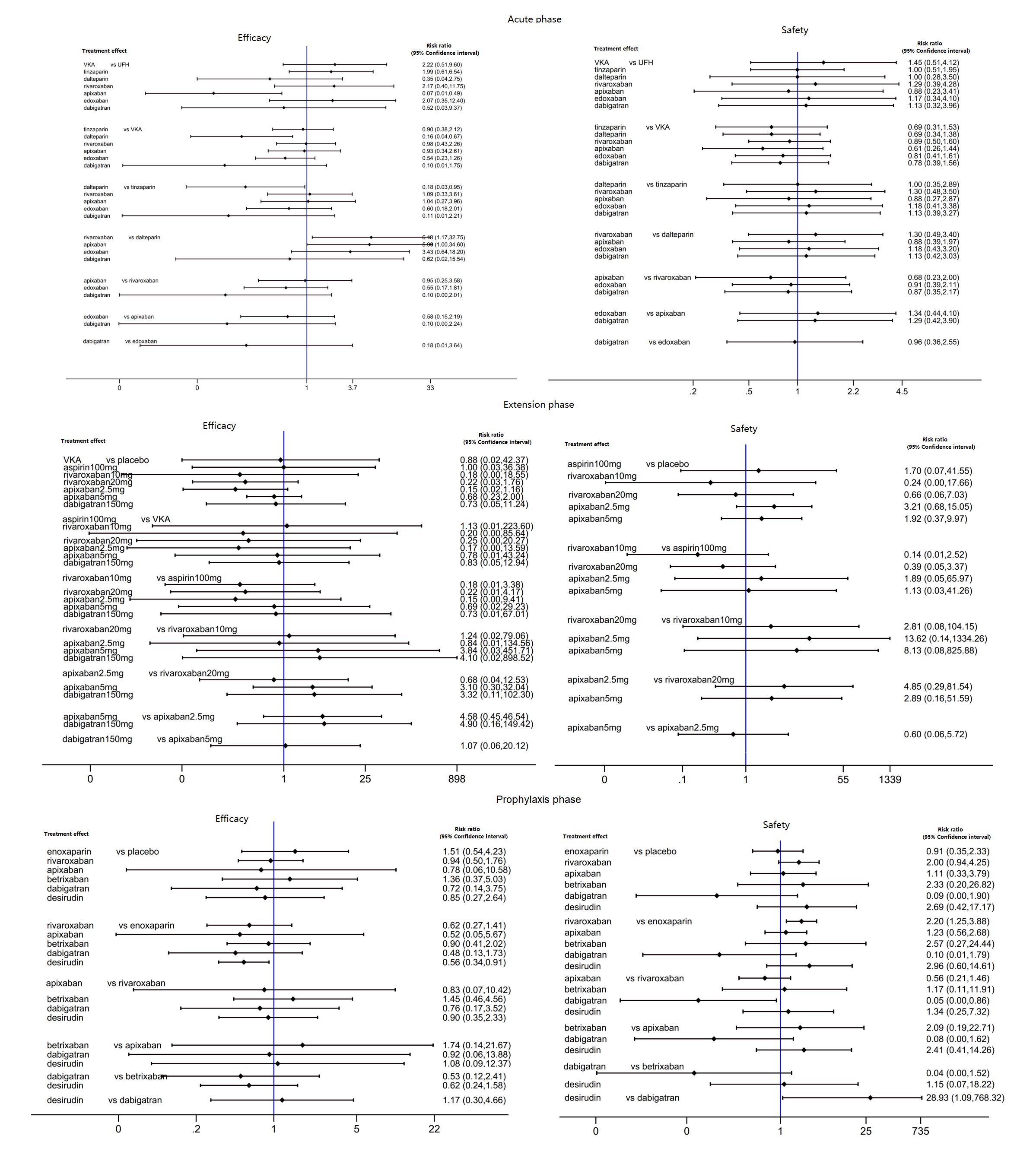
**Supplementary Figure 2. Forest plots of efficacy and safety outcomes among different treatments in VTE patients with renal insufficiency.**


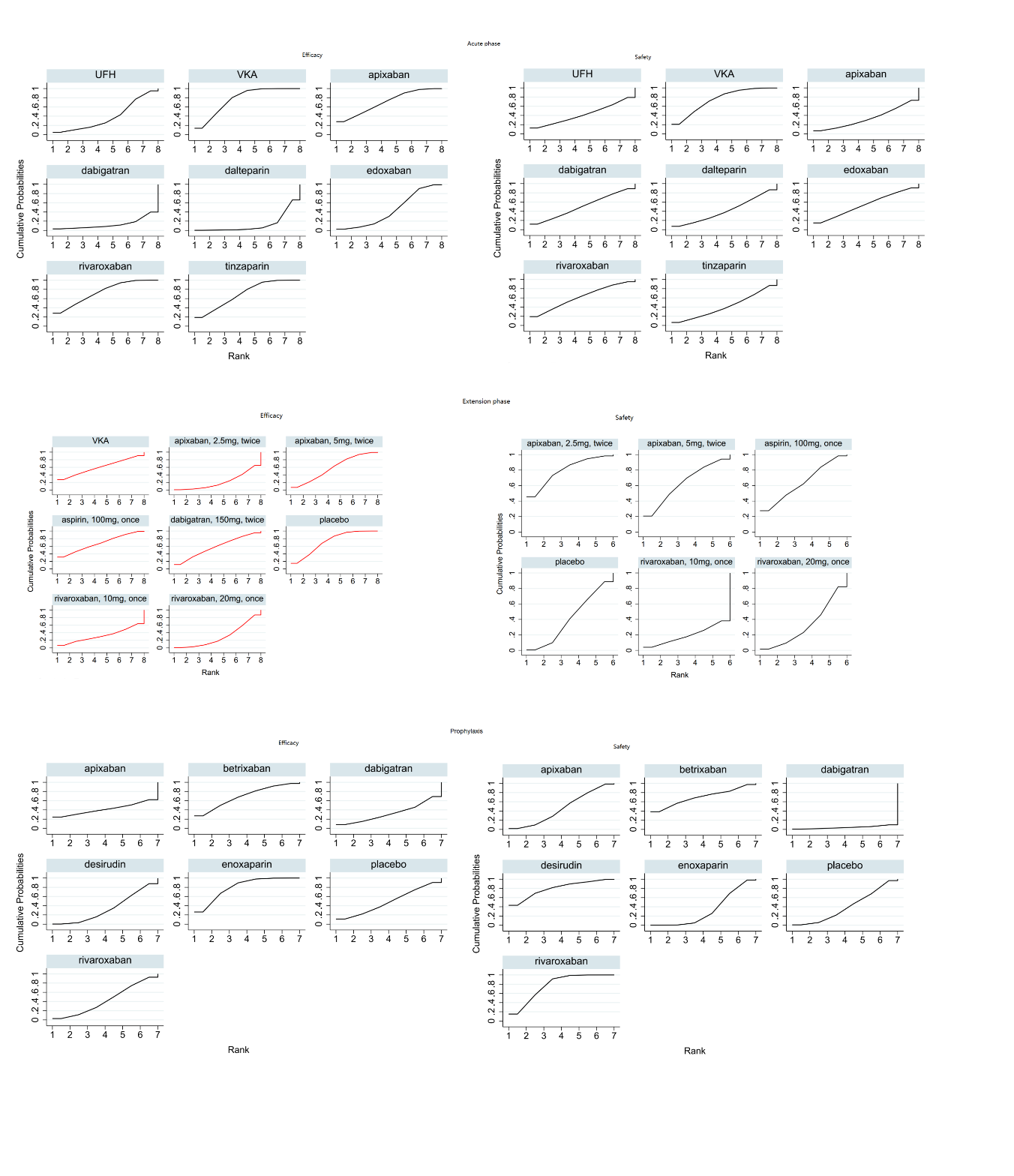


**Supplementary Figure 3. Plots of the surface under the cumulative ranking curves for all treatments in VTE patients with RI by efficacy and safety outcomes.**


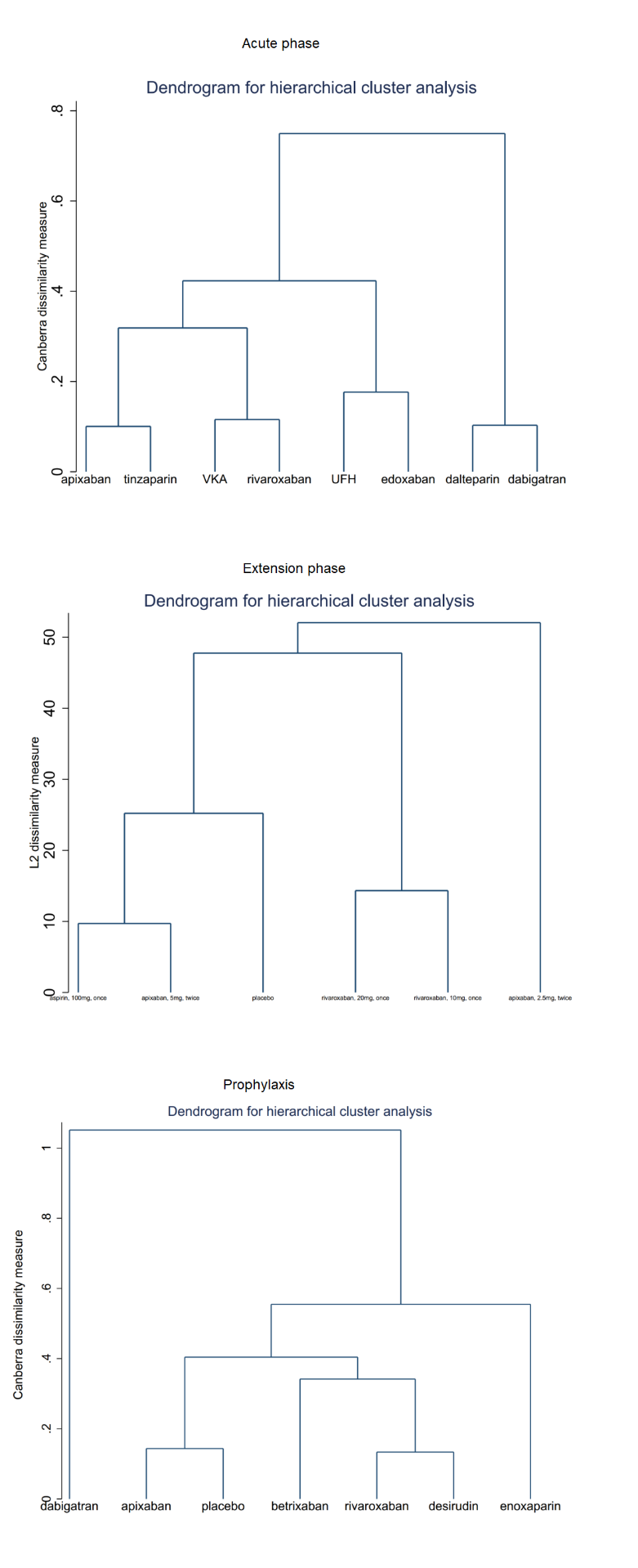


**Supplementary Figure 4. Dendrograms of the hierarchical analysis for all treatments in VTE patients with renal insufficiency by efficacy and safety outcomes.**

**
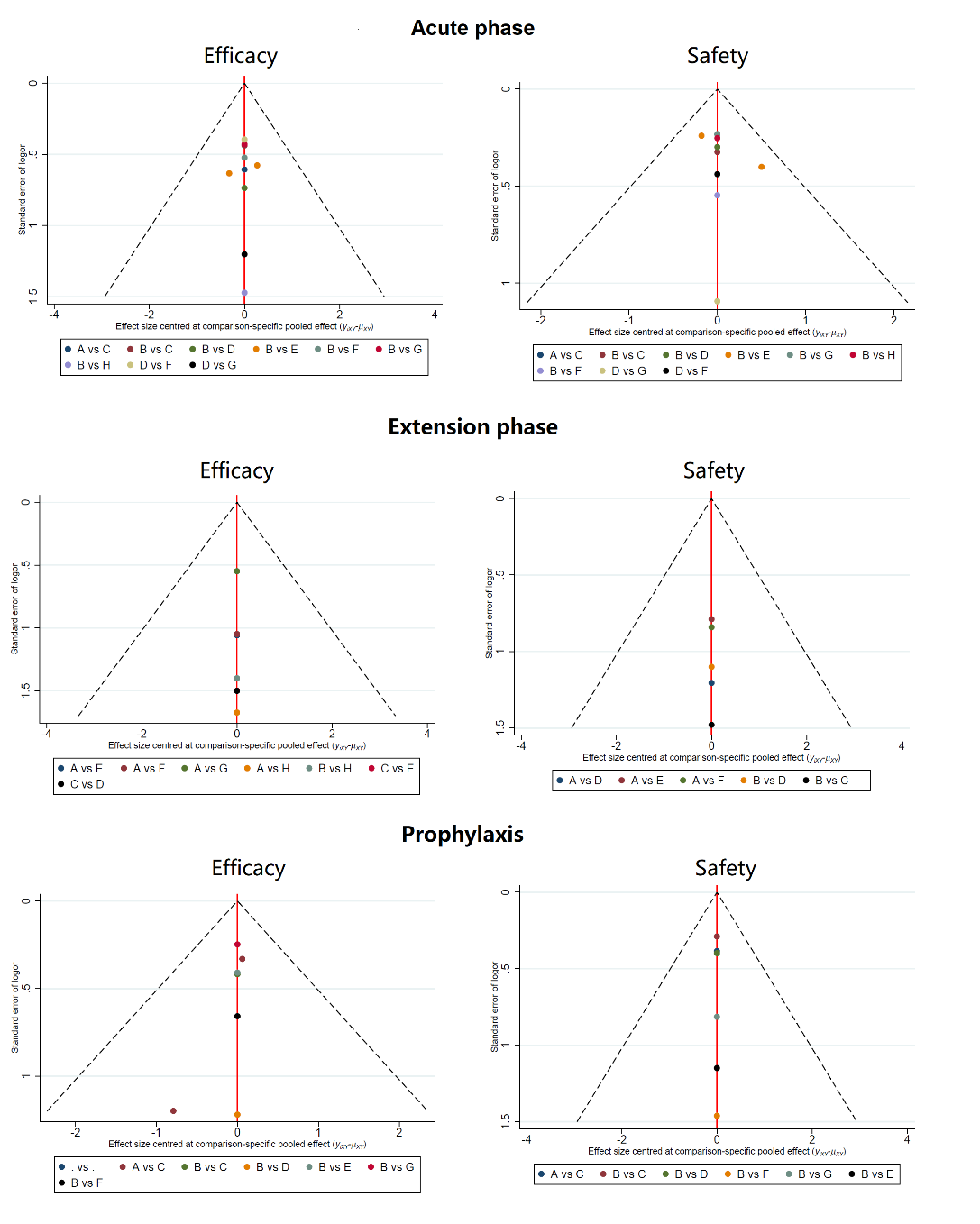
**

**Supplementary Figure 5. Funnel plots of efficacy and safety outcomes in VTE patients with renal insufficiency.**


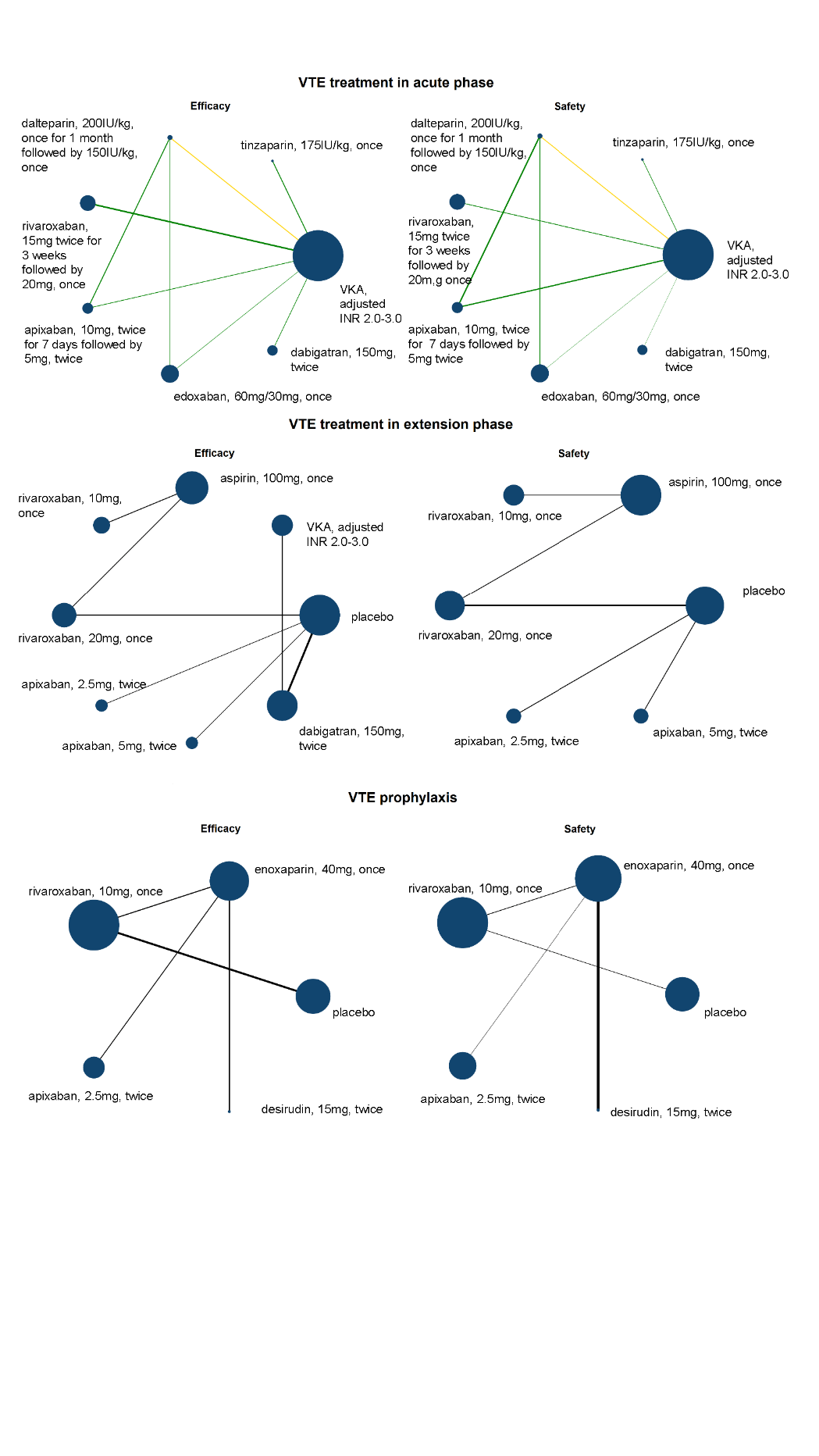


**Supplementary Figure 6. Network plots of VTE treatment network in acute phase, extension phase and prophylaxis for efficacy and safety outcomes of VTE patients without renal insufficiency. (All drugs are present as daily dose and frequency)**

**
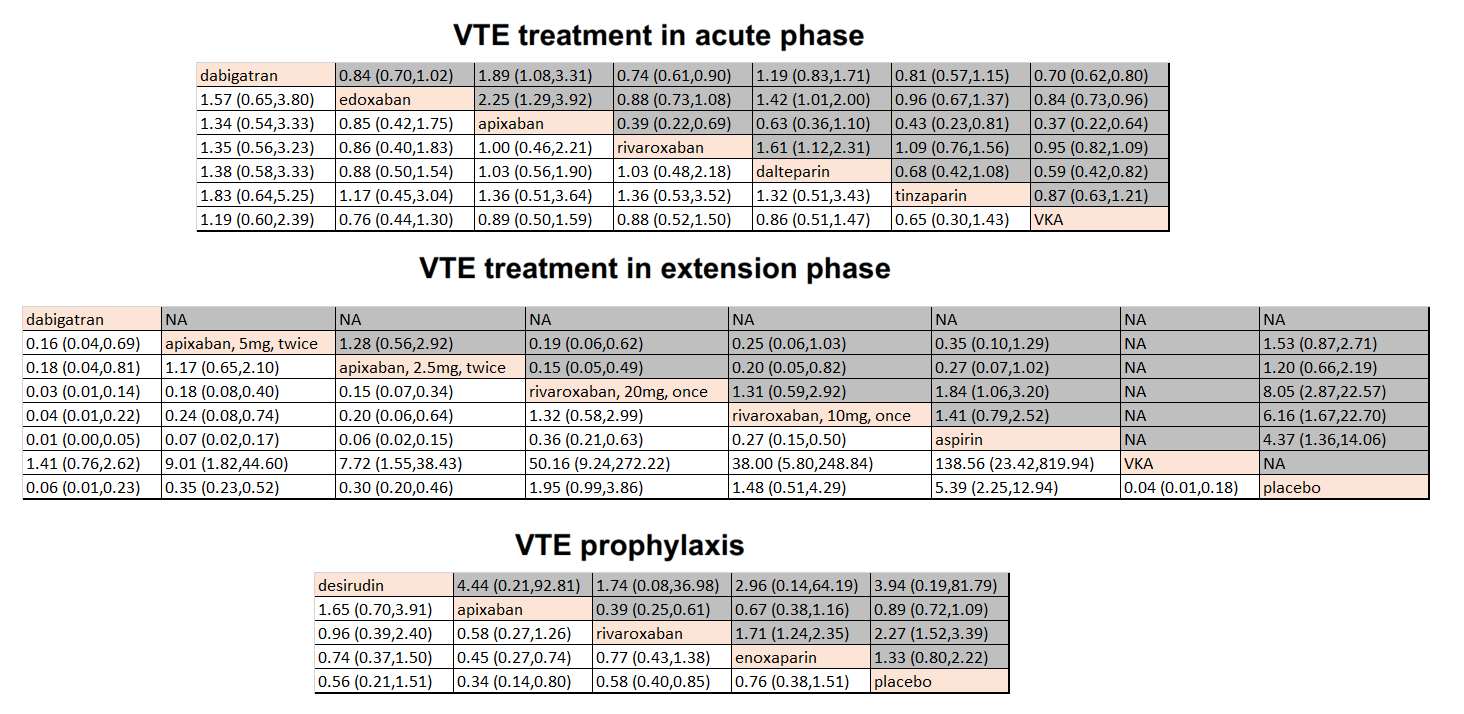
**

**Supplementary Figure 7. The network RR and 95%CI between different treatments in acute phase, extension phase and prophylaxis for efficacy and safety outcomes of VTE patients without renal insufficiency.**

White background (bottom-left): results of efficacy outcome; gray background (top-right): results of safety outcome.


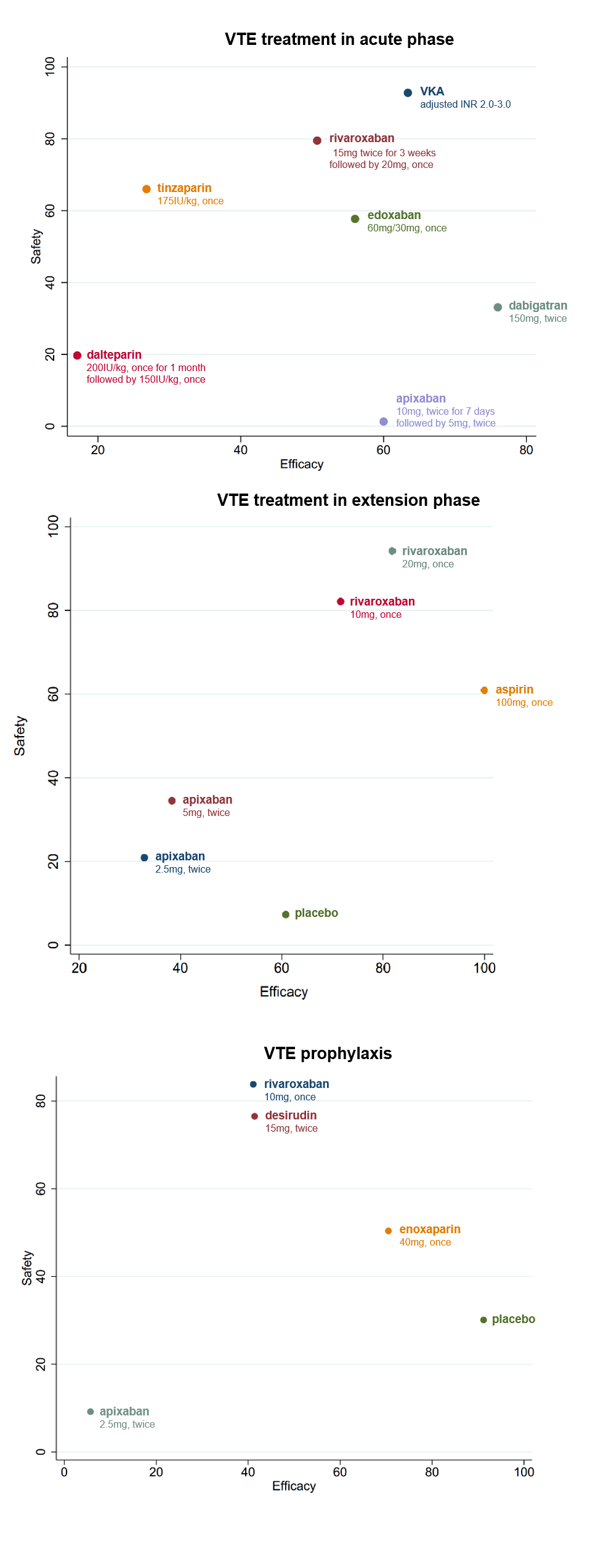


**Supplementary Figure 8. Clustered ranking plot of the different treatments based on cluster analysis for efficacy and safety for VTE patients without renal insufficiency. (All drugs are present as daily dose and frequency)**

**
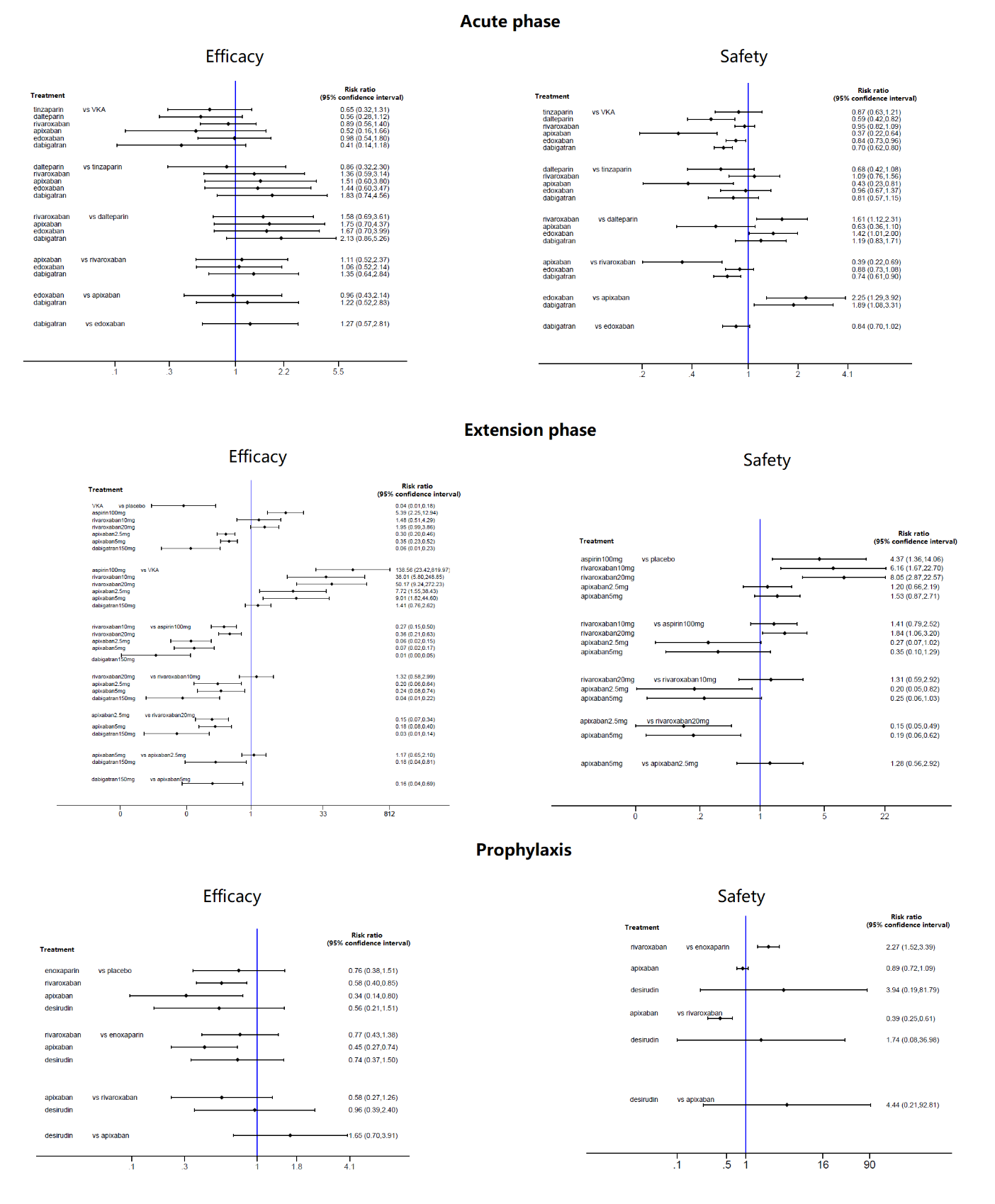
**

**Supplementary Figure 9. Forest plots of efficacy and safety outcomes among different treatments in VTE patients without renal insufficiency.**

**
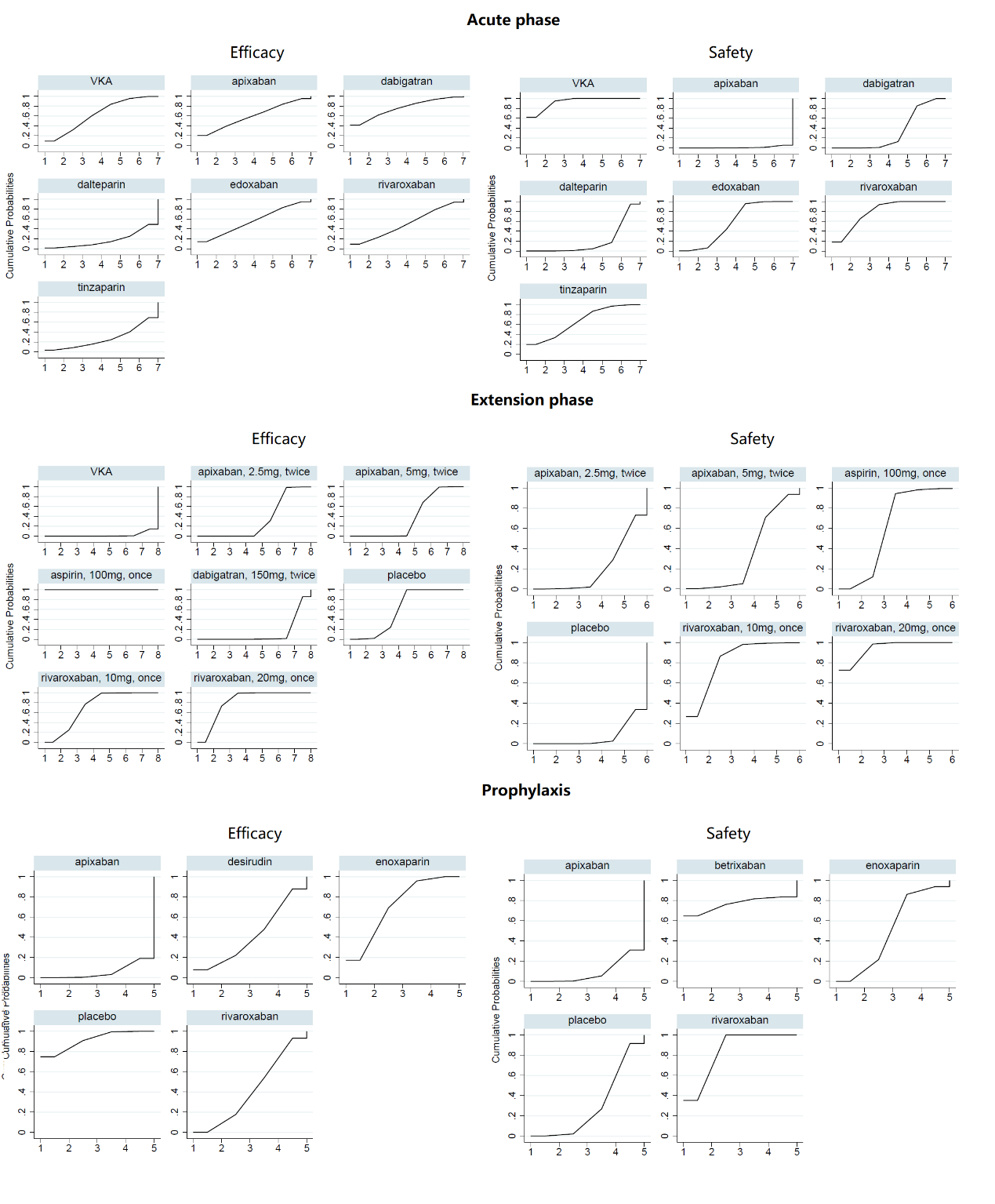
**

**Supplementary Figure 10. Plots of the surface under the cumulative ranking curves for all treatments in VTE patients without renal insufficiency by efficacy and safety outcomes.**

**
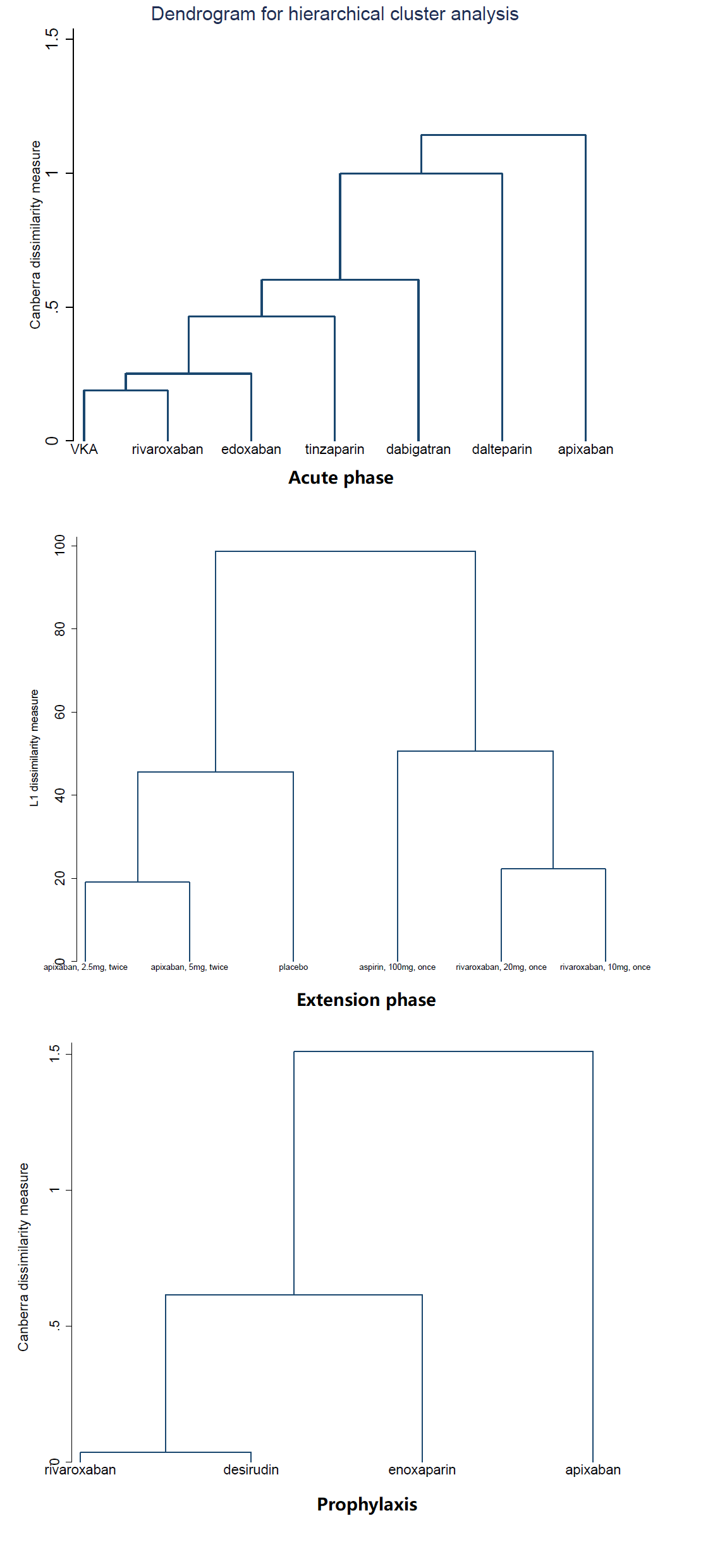
**

**Supplementary Figure 11. Dendrograms of the hierarchical analysis for all treatments in VTE patients without renal insufficiency by efficacy and safety outcomes.**

**
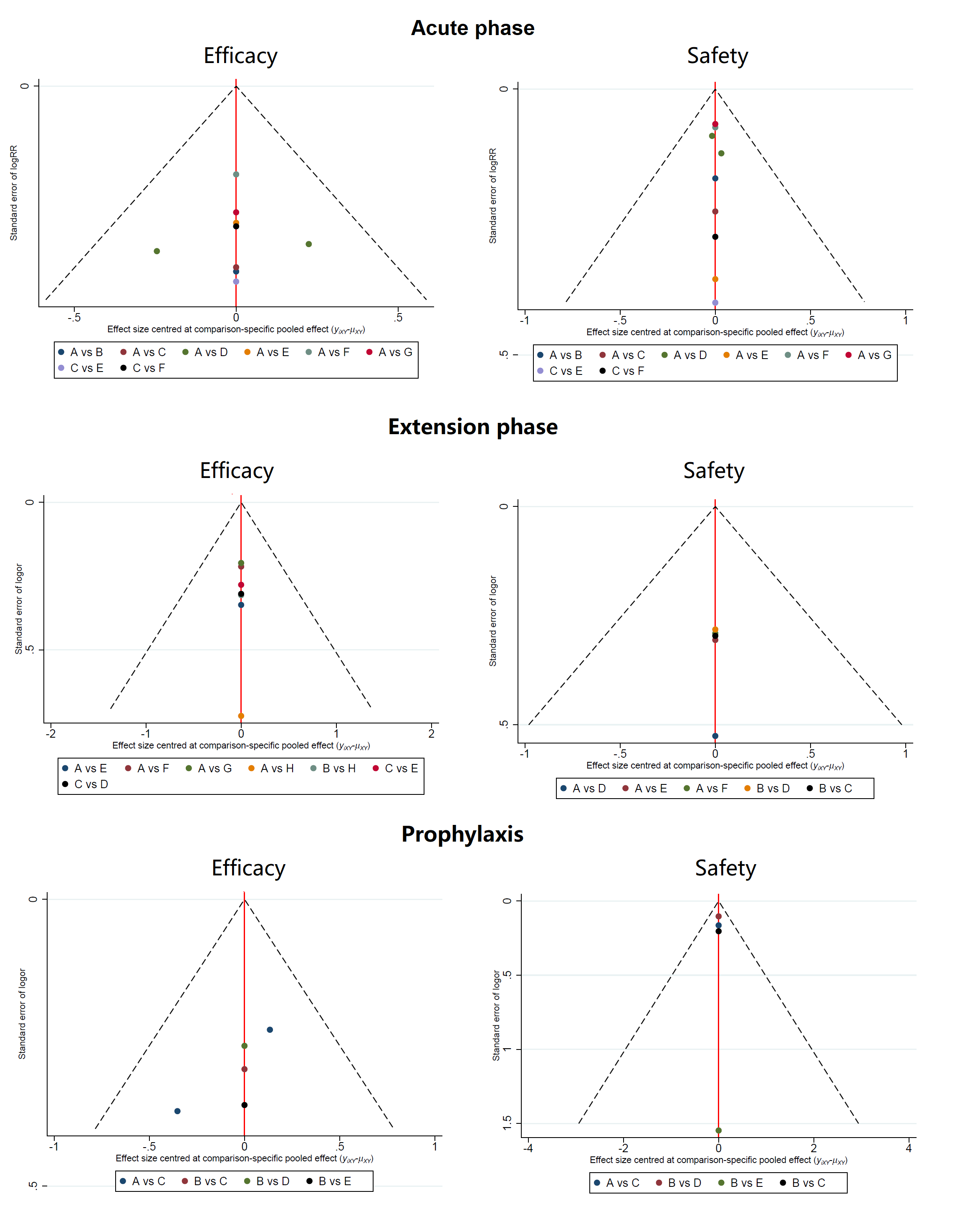
**

**Supplementary Figure 12. Funnel plots of efficacy and safety outcomes in VTE patients without renal insufficiency.**
